# Supplementary material for: CD20/MS4A1 is a mammalian olfactory receptor expressed in a subset of olfactory sensory neurons that mediates innate avoidance of predators
Source: Nat Commun. 2024 Apr 18;15:3360. doi: 10.1038/s41467-024-47698-3 (PMC11026480; doi:10.1038/s41467-024-47698-3)
Supplement: Supplementary file 1 — Supplementary Information [file 41467_2024_47698_MOESM1_ESM.pdf]

Figure S1 related to Figure 1

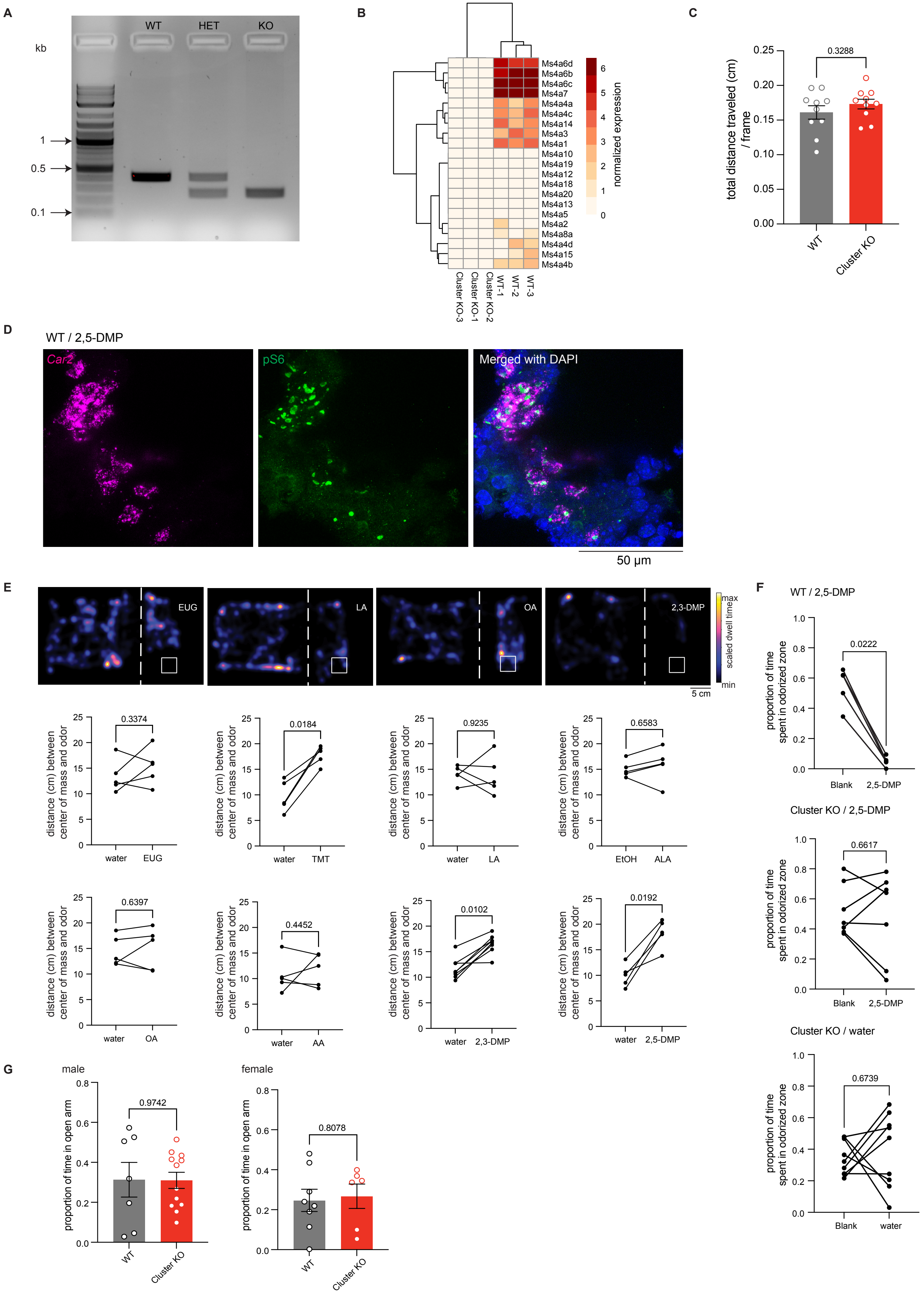

Figure S2 related to Figure 2

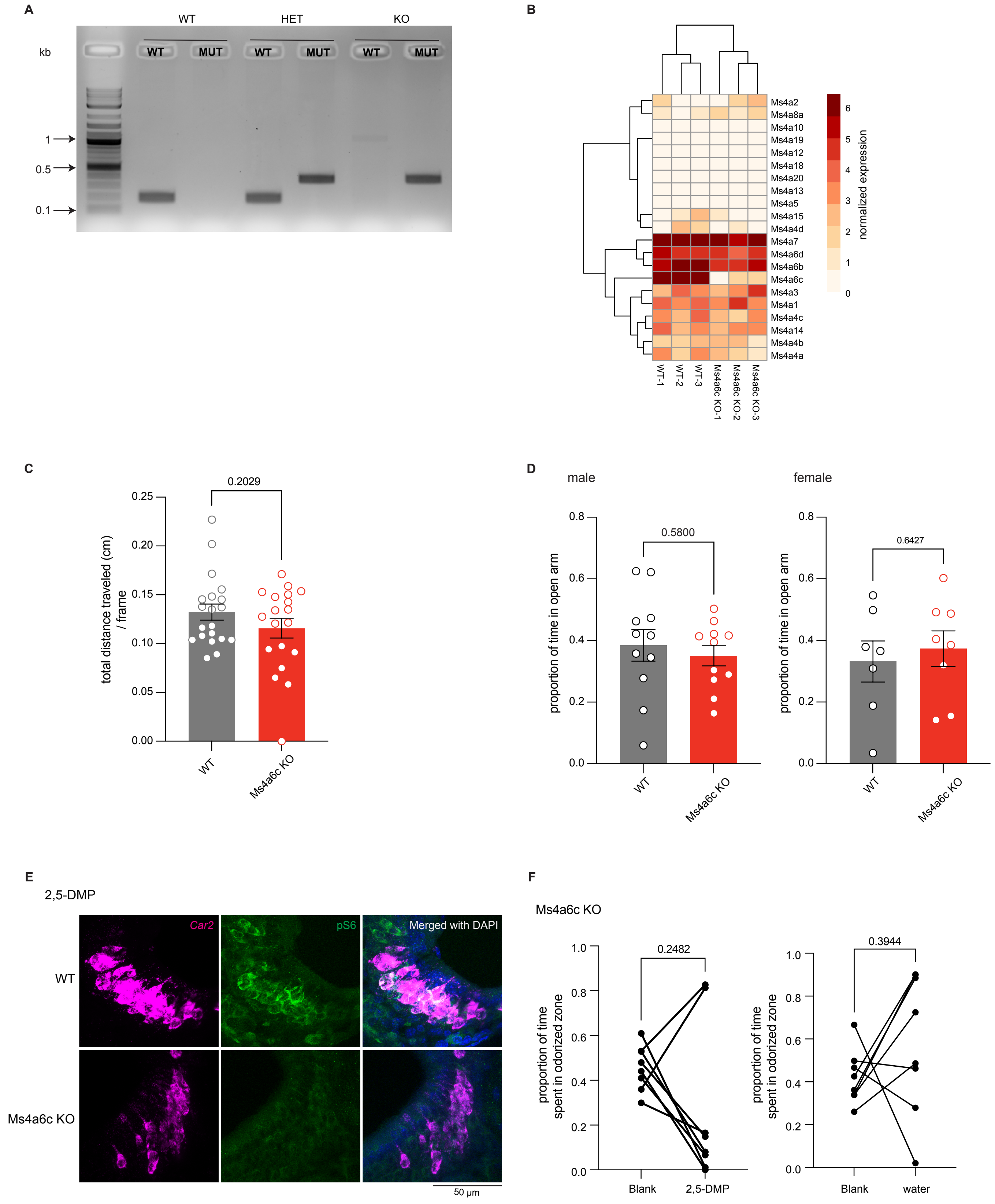

Figure S3 related to Figure 3

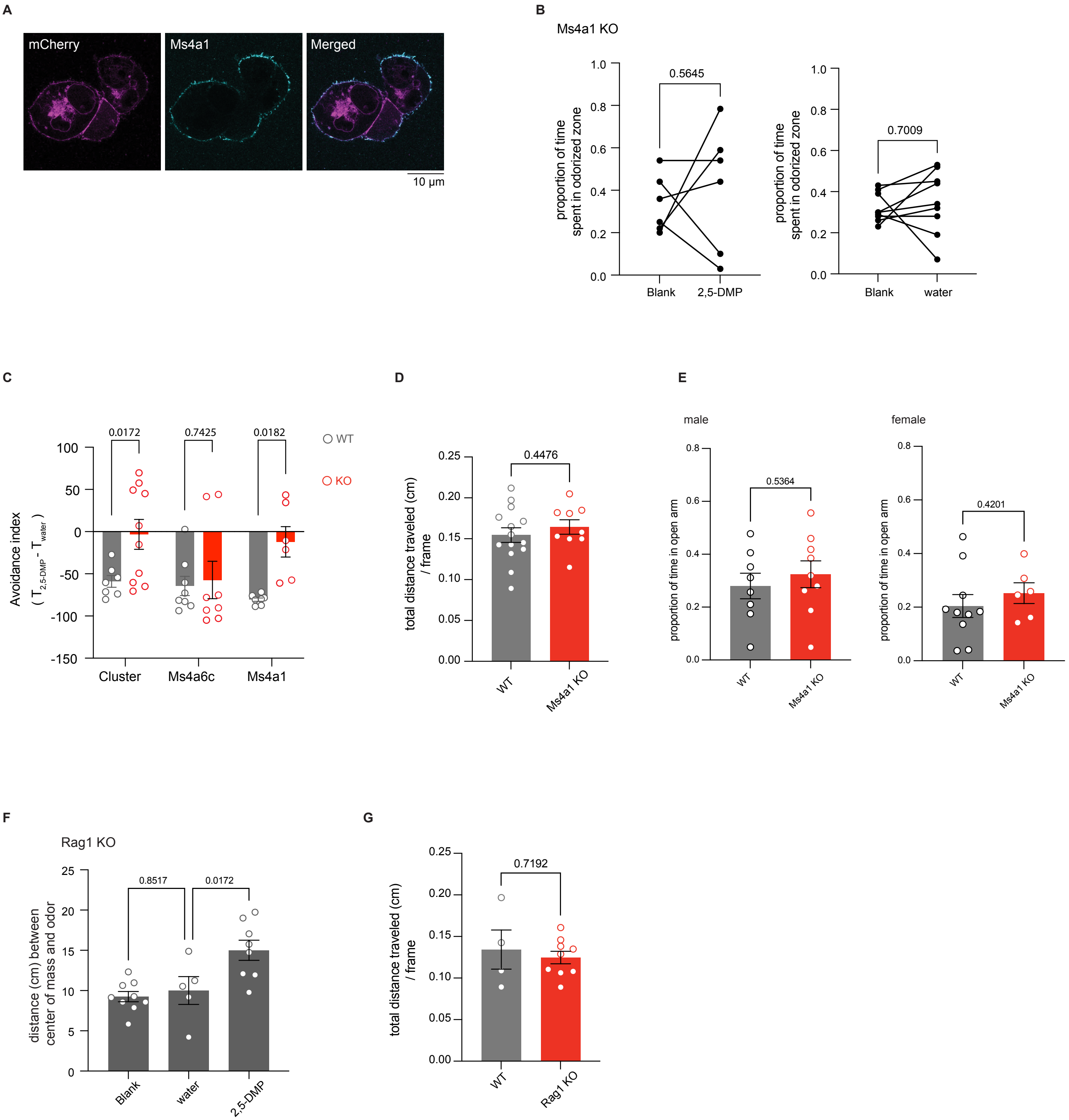

Figure S4 related to Figure 4

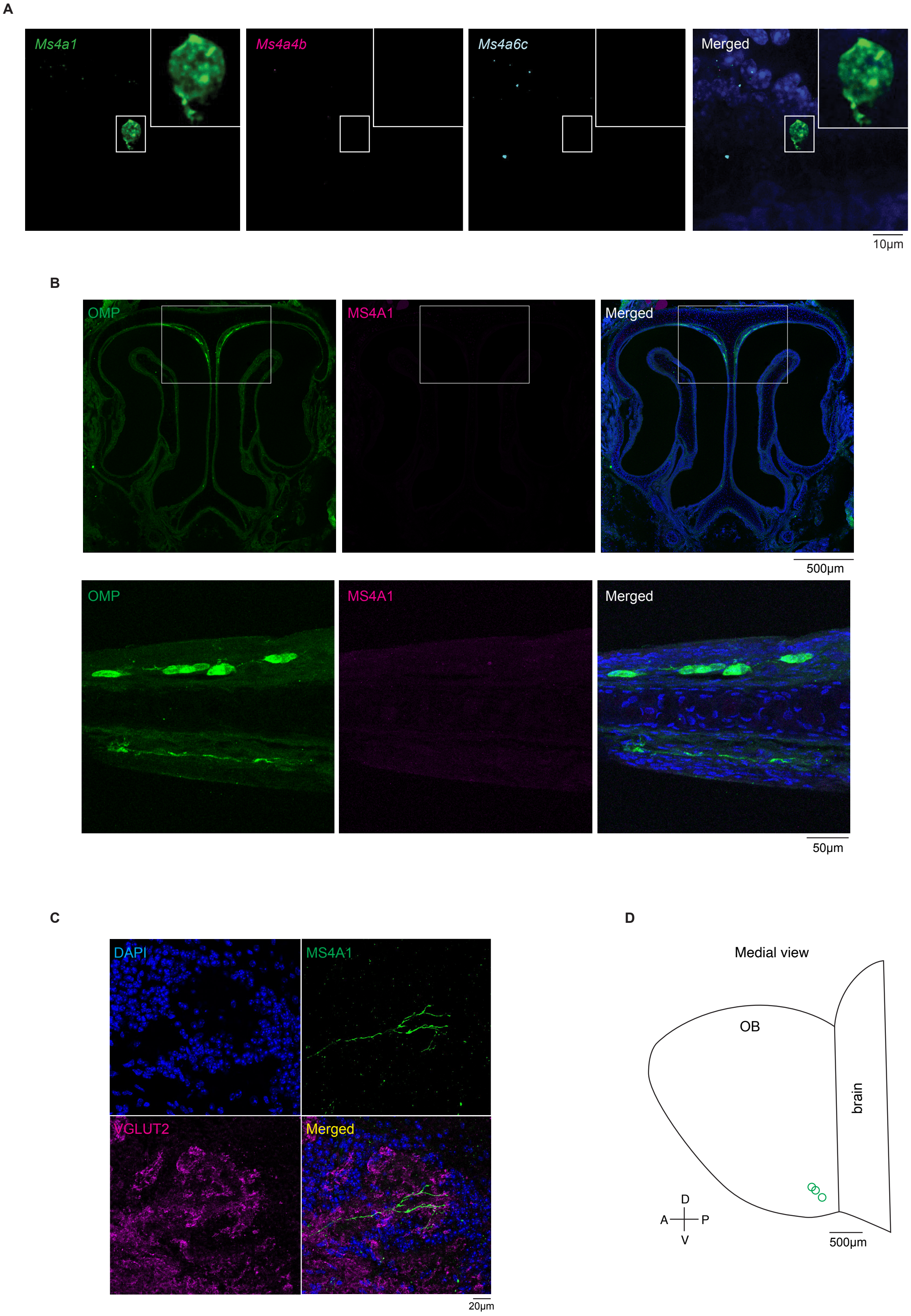

Figure S5 related to Figure 5

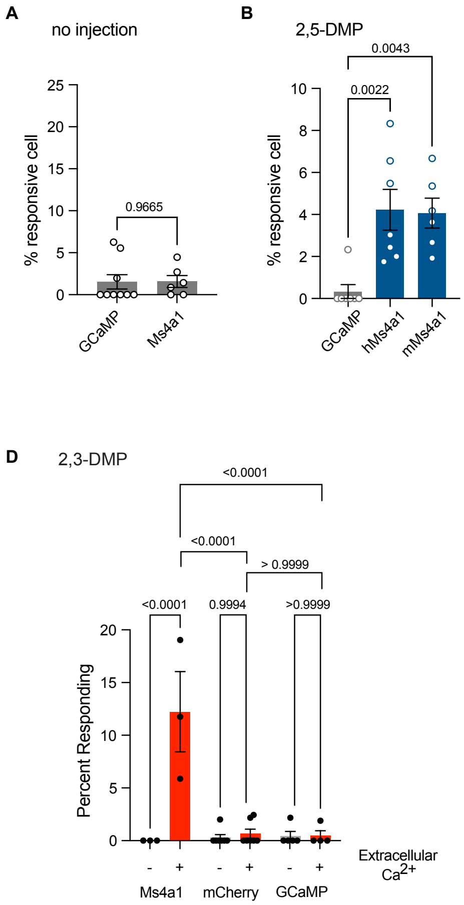

Figure S1 related to Figure 1. Characterization of *Ms4a* cluster knockout mice.

(A) PCR analysis of genomic DNA from wildtype and heterozygous or homozygous Ms4a cluster knockout mice. Wildtype allele: 412 bp; deleted allele: 259 bp. n ≥ 30 mice were tested for each genotype.

(B) Heatmap of the expression of *Ms4a* family member mRNA transcripts detected by RNA sequencing experiments performed on RNA isolated from the main olfactory epithelia of wildtype and *Ms4a* cluster knockout mice.

C) Quantification of the velocity of *Ms4a* cluster knockout mice and their wildtype littermate controls during the odor avoidance assays. Data are presented as mean ± SEM. n = ten mice over five independent experiments (WT), n = ten mice over five independent experiments (Cluster KO). Unpaired Welch two-tailed t-test compared to wildtype mice.

(D) Representative images of the cul-de-sac regions (where necklace cells reside) of the main olfactory epithelia of wildtype mice exposed to 2,5-DMP, co-labeled for the necklace cell marker Car2 (magenta) and the neuronal activity marker phospho-S6 (pSerine240/244) (green).

(E) Heat maps (top panels) of the occupancy of wildtype mice in the odor avoidance chamber in response to the indicated odorants. Small square represents location of odorant, and dashed line demarcates the odor avoidance zone from the rest of the chamber. Scale bar, 5 cm. Bottom panels represent quantification of the avoidance mice exhibit in response to the indicated odors. Paired data were subjected to bootstrapping. A two-tailed t-test was subsequently performed on these data.

(F) The proportion of time spent in the odorized zone was determined for wildtype (top) and *Ms4a* cluster KO mice (middle and bottom) when exposed to the indicated odors. Paired data were subjected to bootstrapping. A two-tailed t-test was subsequently performed on these data.

(G) Quantification of the amount of time male (left panel) and female (right panel) *Ms4a* cluster knockout mice and their wildtype littermate controls spend in open arms in an elevated plus maze assay. Unpaired Welch two-tailed t-test compared to wildtype mice.

Figure S2 related to Figure 2. Characterization of *Ms4a*6c knockout mice.

(A) PCR genotyping analysis of the wildtype and deleted *Ms4a6c* alleles from, wildtype and heterozygous or homozygous *Ms4a6c* knockout mice. Wildtype allele: 104 bp; deleted allele: 400 bp. n ≥ 30 mice were tested for each genotype.

(B) Heatmap of expression of *Ms4a* family member mRNA transcripts detected by RNA sequencing experiments performed on RNA isolated from the main olfactory epithelia of wildtype and *Ms4a6c* knockout mice. The residual *Ms4a6c* transcripts detected in *Ms4a6c* knockout mice all map to 5' UTR regions of the gene, which were not targeted.

(C) Quantification of the velocity of *Ms4a6c* knockout mice and their wildtype littermate controls during odor avoidance assays. Data are presented as mean ± SEM. Unpaired Welch two-tailed t-test compared to wildtype mice.

(D) Quantification of the amount of time male (left panel) and female (right panel) *Ms4a6c* knockout mice and their wildtype littermate controls spend in open arms in an elevated plus maze assay. Data are presented as mean ± SEM. Unpaired Welch two-tailed t-test compared to wildtype mice.

(E) Representative images of the cul-de-sac regions (where necklace cells reside) of the main olfactory epithelia of wildtype and *Ms4a6c* knockout mice exposed to 2,5-DMP, co-labeled for the necklace cell marker Car2 (magenta) and the neuronal activity marker phospho-S6 (pSerine240/244) (green).
(F) The proportion of time spent in the odorized zone was determined for *Ms4a6c* knockout mice in response to the indicated odors. Paired data were subjected to bootstrapping. A two-tailed t-test was subsequently performed on these data.

Figure S3 related to Figure 3. Characterization of *Ms4a*1 knockout mice.
(A) Representative confocal images of HEK293 cells transfected with an N-terminal mCherry-fusion version of MS4A1 (magenta) in which the surface expression of MS4A1 was determined using non-permeabilized staining conditions and an anti-MS4A1 antibody (cyan).
(B) The proportion of time spent in the odorized zone was determined for *Ms4a1* knockout mice in response to the indicated odors. Paired data were subjected to bootstrapping. A two-tailed t-test was subsequently performed on these data.
(C) An avoidance index was calculated for cluster knockout mice, *Ms4a6c* knockout mice, *Ms4a1* knockout mice, and their wildtype littermate controls by subtracting the proportion of time a mouse spent in the odorized zone when exposed to water from the proportion of time a mouse spent in the odorized zone when exposed to 2,5-DMP. For wildtype mice (grey circles), the avoidance index was calculated for n = seven mice over four independent experiments (Cluster), n = eight mice over four independent experiments (Ms4a6c), n = eight mice over four independent experiments (Ms4a1); for knockout mice (red circles), n = ten mice over six independent experiments (Cluster), n = eight mice over three independent experiments (Ms4a6c), n = six mice over three independent experiments (Ms4a1). The data are presented as mean ± SEM. Samples from each group were subjected to bootstrapping. A two-tailed t-test was subsequently performed on these data to compare wildtype and knockout mice.
(D) Quantification of the velocity of *Ms4a1* knockout mice and their wildtype littermate controls during the odor avoidance assays. The data are presented as mean ± SEM. Unpaired Welch two-tailed t-test compared to wildtype mice.
(E) Quantification of the amount of time male (left panel) and female (right panel) *Ms4a1* knockout mice and their wildtype littermate controls spend in open arms in an elevated plus maze assay. The data are presented as mean ± SEM. Unpaired Welch two-tailed t-test compared to wildtype mice.
(F) Quantification of odor avoidance behavior in *Rag1* knockout mice. The distance between the average center of mass of the mouse and the location of odorant was determined for *Rag1* knockout mice in response to the indicated odors. Each circle represents an individual mouse. Data are presented as mean ± SEM, n = nine mice over three independent experiments (Blank), n = five mice over three independent experiments (water), n = eight mice over three independent experiments (2,5-DMP), $^{*}p < 0.05$, Dunnett's multiple comparison test followed by one-way ANOVA test compared to water exposure.
(G) Quantification of the velocity of *Rag1* knockout mice and their wildtype littermate controls during the odor avoidance assays. Data are presented as mean ± SEM. Unpaired Welch two-tailed t-test compared to wildtype mice.

Figure S4 related to Figure 4. Characterization of MS4A1-expressing OSNs.
(A) Determination of whether the non-*Ms4a1 Ms4a* family members, *Ms4a4b* and *Ms4a6c*, are also expressed in MS4A1-expressing cells using single-molecule fluorescent *in situ* hybridization. n = three mice were tested.
(B) Immunostaining of OMP and MS4A1 was performed to determine whether MS4A1 is expressed in the Grueneberg ganglion (highlighted within the white rectangular area) (upper). Representative 63x magnification images obtained from the GG reveal that MS4A1 is not expressed in Grueneberg ganglion neurons. n = three mice were tested.
(C) Immunostaining using antibodies that recognize MS4A1 and VGLUT2 reveals that MS4A1-expressing cells coalesce their axons within a glomerulus in the olfactory bulb. n = three mice were tested.
(D) Schematic representation of the location of the MS4A1glomerulus within the olfactory bulb. iDISCO using antibodies against MS4A1 and VGLUT2 performed on three independent mice reveal that MS4A1-expressing cells converge their axons within a single glomerulus located on the ventral-medial-posterior side of the olfactory bulb. n = three mice were tested.

Figure S5 related to Figure 5. MS4A1 chemoreceptive properties *in vitro*.
(A) Quantification of baseline responses of HEK293 cells expressing MS4A1 protein in the absence of chemical stimulation. The data are presented as mean ± SEM. n = nine wells of cells over six independent experiments (GCaMP), n = six wells of cells over six independent experiments. Unpaired Welch two-tailed t-test compared to GCaMP alone.
(B) Quantification of responses of HEK293 cells expressing either human MS4A1 protein or mouse MS4A1 protein in response to 2,5-DMP. The data are presented as mean ± SEM. n = seven wells of cells over three independent experiments (GCaMP), n = seven wells of cells over three independent experiments (hMs4a1), n = six wells of cells over three independent experiments (mMs4a1). ** $p < 0.01$, Dunnett's test following one-way ANOVA compared to GCaMP alone.
(C) Quantification of responses of HEK293 cells expressing mCherry or MS4A1 to the indicated chemicals as in Figures 5A and 5B. The data are presented as mean ± SEM. For 2,3-DMP, n = 13 wells of cells over three independent experiments (GCaMP), n = 17 wells of cells over three independent experiments (Ms4a1); for 2,5-DMP, n = 13 wells of cells over three independent experiments (GCaMP), n = 12 wells of cells over three independent experiments (Ms4a1); for 2,6-DMP, n = 16 wells of cells over five independent experiments (GCaMP), n = 20 wells of cells over five independent experiments (Ms4a1); for quinoline, n = ten wells of cells over three independent experiments (GCaMP), n = 12 wells of cells over three independent experiments (Ms4a1). $^{*}p < 0.05$, $^{**}p < 0.01$, $^{***}p < 0.001$, Unpaired Welch two-tailed t-test compared to GCaMP alone.
(D) The requirement of extracellular calcium for MS4A1 ligand responses was assessed by stimulating HEK293 cells co-expressing GCaMP6s and either MS4A1 or mCherry or GCaMP6s alone with 2,3-DMP in the presence or absence of extracellular calcium. Data are presented as mean ± SEM. For Ms4a1, n = three wells of cells over three independent experiments (for each indicated condition); for mCherry, n = seven wells of

cells over three independent experiments (for each indicated condition); for GCaMP, n = five wells of cells (without calcium), four wells of cells (with calcium) over three independent experiments. **** $p < 0.0001$, Tuckey's test following one-way ANOVA compared to no extracellular calcium.
