# Supplementary material for: CD20/MS4A1 is a mammalian olfactory receptor expressed in a subset of olfactory sensory neurons that mediates innate avoidance of predators
Source: Nat Commun. 2024 Apr 18;15:3360. doi: 10.1038/s41467-024-47698-3 (PMC11026480; doi:10.1038/s41467-024-47698-3)
Supplement: Supplementary file 3 — Reporting Summary [file 41467_2024_47698_MOESM3_ESM.pdf]

nature portfolio

Corresponding author(s): Paul Greer

Last updated by author(s): March 15, 2024

Reporting Summary

Nature Portfolio wishes to improve the reproducibility of the work that we publish. This form provides structure for consistency and transparency in reporting. For further information on Nature Portfolio policies, see our Editorial Policies and the Editorial Policy Checklist.

Statistics

For all statistical analyses, confirm that the following items are present in the figure legend, table legend, main text, or Methods section.

n/a	Confirmed	
☐	☒	The exact sample size (*n*) for each experimental group/condition, given as a discrete number and unit of measurement
☐	☒	A statement on whether measurements were taken from distinct samples or whether the same sample was measured repeatedly
☐	☒	The statistical test(s) used AND whether they are one- or two-sided<br>*Only common tests should be described solely by name; describe more complex techniques in the Methods section.*
☒	☐	A description of all covariates tested
☐	☒	A description of any assumptions or corrections, such as tests of normality and adjustment for multiple comparisons
☐	☒	A full description of the statistical parameters including central tendency (e.g. means) or other basic estimates (e.g. regression coefficient) AND variation (e.g. standard deviation) or associated estimates of uncertainty (e.g. confidence intervals)
☐	☒	For null hypothesis testing, the test statistic (e.g. *F*, *t*, *r*) with confidence intervals, effect sizes, degrees of freedom and *P* value noted<br>*Give P values as exact values whenever suitable.*
☒	☐	For Bayesian analysis, information on the choice of priors and Markov chain Monte Carlo settings
☒	☐	For hierarchical and complex designs, identification of the appropriate level for tests and full reporting of outcomes
☒	☐	Estimates of effect sizes (e.g. Cohen's *d*, Pearson's *r*), indicating how they were calculated

Our web collection on statistics for biologists contains articles on many of the points above.

Software and code

Policy information about availability of computer code

Data collection	DolphinNext (https://dolphinnext.umassmed.edu/)<br>STAR (v2.6.1)<br>RSEM (v1.3.1)<br>R (4.0.0 or higher)<br>python (3.7.6 or higher)<br>ezTrack(v1.2)<br>DESeq2 (1.30.1)<br>ggplot2(3.3.4 or higher)<br>Imaris x64 software (8.0.1 or higer)
Data analysis	ll the scripts used for this study can be found at: https://github.com/Greerlab/CD20_2023_paper

For manuscripts utilizing custom algorithms or software that are central to the research but not yet described in published literature, software must be made available to editors and reviewers. We strongly encourage code deposition in a community repository (e.g. GitHub). See the Nature Portfolio guidelines for submitting code & software for further information.

Data

Policy information about availability of data

All manuscripts must include a data availability statement. This statement should provide the following information, where applicable:

- Accession codes, unique identifiers, or web links for publicly available datasets
- A description of any restrictions on data availability
- For clinical datasets or third party data, please ensure that the statement adheres to our policy

All RNA sequencing data described in this manuscript are deposited at GEO accession GSE240378.
Gencode database (Release M25, GRCm38.p6) was used for building the transcriptome reference

Research involving human participants, their data, or biological material

Policy information about studies with human participants or human data. See also policy information about sex, gender (identity/presentation), and sexual orientation and race, ethnicity and racism.

Reporting on sex and gender	N.A.. No human related study involved
Reporting on race, ethnicity, or other socially relevant groupings	N.A.. No human related study involved
Population characteristics	N.A.. No human related study involved
Recruitment	N.A.. No human related study involved
Ethics oversight	N.A.. No human related study involved

Note that full information on the approval of the study protocol must also be provided in the manuscript.

Field-specific reporting

Please select the one below that is the best fit for your research. If you are not sure, read the appropriate sections before making your selection.

☒ Life sciences ☐ Behavioural & social sciences ☐ Ecological, evolutionary & environmental sciences

For a reference copy of the document with all sections, see nature.com/documents/nr-reporting-summary-flat.pdf

Life sciences study design

All studies must disclose on these points even when the disclosure is negative.

Sample size	A sample size of 3 biological replicates were selected for bulk RNAseq. No statistical tests were used to predetermine sample size, but our sample sizes were similar to those reported in previous publication (Saraiva, L., Ibarra-Soria, X., Khan, M. et al. Hierarchical deconstruction of mouse olfactory sensory neurons: from whole mucosa to single-cell RNA-seq. (2015) Sci Rep 5, 18178). For pS6 staining, a sample size of at least 50 individual cells from each of the 3 biological replicates was selected. No statistical tests were used to predetermine sample size, but our sample sizes were similar to those reported in previous publication (Greer PL, Bear DM. et al. (2016) A family of non-GPCR chemosensors defines an alternative logic for mammalian olfaction. Cell, 165: 1734-48). For Calcium imaging, a sample size of at least 50 individual cells from each of the 3 biological replicates was selected. No statistical tests were used to predetermine sample size, but our sample sizes were similar to those reported in previous publication (Greer PL, Bear DM. et al. (2016) A family of non-GPCR chemosensors defines an alternative logic for mammalian olfaction. Cell, 165: 1734-48). For behavioral assay, a sample size of at least 6 biological replicates were selected, No statistical tests were used to predetermine sample size, but our sample sizes were similar to those reported in previous publication (Kobayakawa, K., Kobayakawa, R., Matsumoto, H. et al. Innate versus learned odour processing in the mouse olfactory bulb. (2007) Nature 450, 503–508).
Data exclusions	In the behavioral assay, the data of the inactive animals were excluded (locomotion < 10 cm/30 mins during the habituation).
Replication	At least three independent biological replicates were performed for each experiment. All replicates were successfully done and included.
Randomization	N.A. No randomization was needed or performed in this study. because only 1 stimulus was used at a time.
Blinding	The researcher was blinding to the genotype and treatment in quantifying the calcium imaging and pS6 staining experiments to ensure the unbiased quantification. The behavioral assay was analyzed using an automated pipeline independent of genotype and treatment, no human input involved.

Reporting for specific materials, systems and methods

We require information from authors about some types of materials, experimental systems and methods used in many studies. Here, indicate whether each material, system or method listed is relevant to your study. If you are not sure if a list item applies to your research, read the appropriate section before selecting a response.

Materials & experimental systems

n/a	Involved in the study
☐	☒ Antibodies
☐	☒ Eukaryotic cell lines
☒	☐ Palaeontology and archaeology
☐	☒ Animals and other organisms
☒	☐ Clinical data
☒	☐ Dual use research of concern
☒	☐ Plants

Methods

n/a	Involved in the study
☒	☐ ChIP-seq
☒	☐ Flow cytometry
☒	☐ MRI-based neuroimaging

Antibodies

Antibodies used

rabbit anti-phospho-S6 ribosomal protein (Serine240/244) (1:100, Cell Signaling Technologies, #2215),
rabbit anti-phospho-S6 ribosomal protein (Serine244/247) (1:150, Invitrogen, #44-923G),
rabbit anti-MS4A1/CD20 (1:250 for immunostaining, 1:100 for iDISCO, Cell Signaling Technology, #98708),
rabbit anti-MS4A1 (1:200, MyBioSource, #MBS2051903),
goat anti-MS4A1 (1:50, Santa Cruz Biotechnology, #sc-7735),
rat anti-MS4A1 (1:100, LifeSpan Biosciences, #LS-C107163-100),
guinea pig anti-458 VGLUT2 (1:500, SYSY, #135404),
rabbit anti-NeuN (1:500, Abcam, #ab104225),
rabbit anti-KI18 (1:500, Abcam, #ab52948),
rabbit anti-KI17 (1:500, Abcam, #ab53707),
goat anti-NeuroD1 (1:50, R&D Systems, #AF2746),
goat anti-OMP (1:1000, Wako Chemicals, #544-10001-WAKO),
rabbit anti-CNGA2 (1:200, Alomone Labs, #APC-045),
rabbit anti-PDE2A (1:500, FabGennix, #PD2A-101AP),
alpaca anti-rabbit Alexa488 (1:333, Jackson Immunoresearch, #611-545-215),
alpaca anti-rabbit rhodamine red X (RRX) (1:333, Jackson Immunoresearch, #611-295-215),
goat anti-rabbit-Alexa647 (1:333, Invitrogen, #A-21245),
bovine anti-goat Alexa488 (1:333, Jackson Immunoresearch, #805-545-180),
bovine anti-goat Alexa647 (1:333, Jackson Immunoresearch, #805-605-180),
goat anti-rat Alexa488 (1:333, Invitrogen, #A-11006),
donkey anti-rat RRX (1:333, Jackson Immunoresearch, #712-295-153),
goat anti-guinea pig Alexa647 (1:333, Invitrogen, #A21450).

Validation

To determine the specificity of the anti-MS4A1 antibodies in the mouse olfactory epithelium and olfactory bulb, we performed immunohistochemical analysis on olfactory epithelium and olfactory bulbs isolated from MS4A1 knockout mice to make sure that no antibody signals in the region of interest was observed.
To determine the specificity of all of the secondary antibodies in the mouse olfactory epithelium and olfactory bulb, we performed immunohistochemical analyses on olfactory epithelium and olfactory bulbs without primary antibodies and no staining was observed.

The rabbit anti-phospho-S6 ribosomal protein (Cell Signaling Technologies, #2215) has been shown to label the mouse neurons in activity-dependent manner (DOI: https://doi.org/10.1038/nn.3734).
The rabbit anti-phospho-S6 ribosomal protein (Invitrogen, #44-923G) has been shown to label the mouse neurons in activity-dependent manner (DOI:https://doi.org/10.1016/j.cmet.2021.03.001).
The guinea pig anti-458 VGLUT2 (1:500, SYSY, #135404) has been shown to specifically labeled the mouse olfactory glomeruli (https://doi.org/10.1016/j.mcn.2018.01.010).
The rabbit anti-NeuN (1:500, Abcam, #ab104225) has been used as a pan-neuron marker in many studies (https://www.biocompare.com/9776-Antibodies/1642758-FOX3-antibody/#citations).
The rabbit anti-KI18 (1:500, Abcam, #ab52948) has been used as a sustentacular cell marker in mouse olfactory system (https://doi.org/10.1523/JNEUROSCI.1708-15.2015).
The rabbit anti-KI17 (1:500, Abcam, #ab53707) has been used as a horizontal basal cell marker in olfactory system (https://doi.org/10.1002/lary.21856).
The goat anti-NeuroD1 (1:50, R&D Systems, #AF2746) has been used as a globose basal cell marker in mouse olfactory system (https://doi.org/10.1002/lary.21856).(https://doi.org/10.1016/j.stem.2017.09.008).
The goat anti-OMP (1:1000, Wako Chemicals, #544-10001-WAKO) has been used as a olfactory sensory neuron marker in many studies (https://labchem-wako.fujifilm.com/us/product/detail/WAKUWAK544-10001-WAKO.html).
The rabbit anti-CNGA2 (1:200, Alomone Labs, #APC-045) has been used to specifically detect the CNGA2 expression in the mouse olfactory system (https://doi.org/10.1523/JNEUROSCI.2527-16.2016).
The rabbit anti-PDE2A (1:500, FabGennix, #PD2A-101AP) has been used as a GC-D neuron marker in the mouse olfactory system(https://doi.org/10.1093/chemse/bjaa027)

Eukaryotic cell lines

Policy information about cell lines and Sex and Gender in Research

Cell line source(s)	Human embryonic kidney 293 (HEK293) cells: source: ATCC, #CRL-3216<br>A20 cells: source: ATC, #TIB-208
Authentication	All cell lines were authenticated
Mycoplasma contamination	Mycoplasma were not detected in all cell lines
Commonly misidentified lines<br>(See ICLAC register)	no misidentified line were used in this study.

Animals and other research organisms

Policy information about studies involving animals; ARRIVE guidelines recommended for reporting animal research, and Sex and Gender in Research

Laboratory animals	This study used several mouse lines (mus musculus) following federal guidelines (12 hr. light/ 12 hr. dark cycle, 20-23°C, 30-70% humidity)<br>C57BL/6J - Male and female adult mice were used, 2~4 month old.<br>Ms4a1 knockout - Male and female adult mice were used, 2~4 month old.<br>Ms4a6c knockout - Male and female adult mice were used, 2~4 month old.<br>Ms4a cluster kncokout - Male and female adult mice were used, 2~4 month old.<br>Rag1 kncokout - Male and female adult mice were used, 2~4 month old.
Wild animals	Study did not involve wild animals.
Reporting on sex	In all experiments, data were collected from both gender with equal ratio.
Field-collected samples	Study did not involve field-collected samples.
Ethics oversight	All animal care and use procedures were followed in accordance with federal guidelines and approved by the University of Massachusetts Medical School Institutional Animal Care and Use Committee (Protocol 202100120)

Note that full information on the approval of the study protocol must also be provided in the manuscript.

Plants

Seed stocks	N.A.
Novel plant genotypes	N.A.
Authentication	N.A.
